# Molecular Authentication of the Medicinal Species of *Ligusticum* (Ligustici Rhizoma et Radix, “Gao-ben”) by Integrating Non-coding Internal Transcribed Spacer 2 (ITS2) and Its Secondary Structure

**DOI:** 10.3389/fpls.2019.00429

**Published:** 2019-04-09

**Authors:** Zhen-wen Liu, Yu-zhen Gao, Jing Zhou

**Affiliations:** ^1^CAS Key Laboratory for Plant Diversity and Biogeography of East Asia, Kunming Institute of Botany, Chinese Academy of Sciences, Kunming, China; ^2^School of Pharmaceutical Sciences and Yunnan Key Laboratory of Pharmacology for Natural Products, Kunming Medical University, Kunming, China

**Keywords:** apiaceae, DNA barcoding, Ligustici Rhizoma et Radix, ITS2, secondary structures

## Abstract

Ligustici Rhizoma et Radix (LReR), an important Chinese medicine known as “Gao-ben,” refers to *Ligusticum sinense* Oliv. or *Ligusticum jeholense* Nakai et Kitag. However, a number of other species are commonly sold as “Gao-ben” in the herbal medicine market, which may result in a series of quality control problems and inconsistent therapeutic effects. The “Gao-ben” is commonly sold sliced and dried, making traditional identification methods difficult. Here, the mini barcode ITS2 region was examined on 68 samples representing LReR and 7 potential adulterant or substitute species. The results showed 100% success rates of PCR and sequencing and the existence of a barcoding gap. The neighbor-joining (NJ) tree indicated that all the tested samples could be exactly identified. The ITS2 secondary structure revealed a clear difference between true “Gao-ben” and three adulterant species. We therefore recommend the use of ITS2 as a mini barcode for distinguishing between closely or distantly related plant species that may be used in Chinese medicine.

## Introduction

Apiaceae, the 16th-largest flowering plant family, comprises more than 3,540 species in 446 genera (Mabberley, 1997). It is a well-known and economically important plant family in medicine, spices, vegetables and ornamental gardening. The roots and rhizomes of *Ligusticum sinense* Oliv. or *Ligusticum jeholense* Nakai et Kitag form a widely used traditional Chinese medicine, known as “Ligustici Rhizoma et Radix” (LReR), or “Gao-ben” in Chinese (Commission, 2015). It is commonly used to treat colds, trapped wind, headaches and rheumatic arthralgia (Commission, 2015). It has been reported to exhibit many beneficial properties such as analgesic, antipyretic, anti-inflammatory and anticonvulsive activities, plus antimicrobial and antioxidant effects (Wang et al., 2011). As a result, this herb has attracted more and more attention in the medical field, and have been widely used in clinical therapies.

However, LReR is easily confused with other herbs, causing potential mistakes in treatment. Certain species that are closely related or morphologically similar to LReR are frequently used as local remedies in various regions due to geographical and historical factors. For instance, *Meeboldia yunnanensis* (H. Wolff) Constance & F. T. Pu, *Ligusticum delavayi* Franchet and *Ligusticum pteridophyllum* Franchet are often used in folk medicine in southwestern China, but their function and efficacy are not quite the same (Li et al., 2001). Additionally, *Conioselinum vaginatum* (Spreng.) Thell., *Ligusticum tenuissimum* (Nakai) Kitagawa, *Sium suave* Walter and *Ligusticum acuminatum* Franch. are also sold as “Gao-ben” in medicinal markets. Using morphology-based or chemical identification methods to identify Rhizoma et Radix herbs is difficult, especially when the plant is sliced and dried, as they often are for selling (Hon et al., 2003; Joshi et al., 2004; Xue et al., 2006; Mishra et al., 2016). Therefore, a simple, inexpensive and effective method for distinguishing between the above species is urgently needed.

DNA barcoding is a new taxonomic method that uses one or a few short, standard genomic DNA region(s) for rapid, reliable and effective species identification (Floyd et al., 2002; Hebert et al., 2003). For identifying traditional herbal medicines, however, the commonly used three-barcode system has not always been effective, because matK and *rbc*L are difficult to amplify, especially from powdered products where DNA degradation is very common. The internal transcribed spacer 2 (ITS2) region of nuclear ribosomal DNA might be a promising standardized region to barcode medicinal plants, due to its relatively short length, consistent performance in distinguishing closely related species, and ease of amplification with a single set of universal primers (Chen et al., 2010). In cells, ITS2 has conserved nucleotide motifs which play a role in forming a three dimensional structure, and hence transformation into a functional complex before conversion to a mature rRNA (Hall, 1999; Lalev and Nazar, 1999). Compared with previous sequence alignments based only on nucleotide similarity, the ITS2 conserved nucleotide motifs permit multiple sequence alignments, from which a more homologous overall alignment can be generated (Kjer, 1995; Coleman and Mai, 1997). Additionally, the secondary structure is maintained by the mutual between base-pairs that are canonical (GC, AU), non-canonical stable (GU) and unstable (AC) (Elgavish et al., 2001; Leontis and Westhof, 2001). Theses paired and unpaired ITS2 structures provide extra molecular morphological features that can greatly improve taxonomic classification (Telford et al., 2005; Keller et al., 2010).

In this study, we use ITS2 sequence and secondary structure information to determine whether genuine “Gao-ben” can be distinguished from the most commonly used adulterants and substitutes. We also discuss the possibility that ITS2 might play a regulatory role in the herbal medicine market in the future.

## Materials and Methods

### Plant Materials

A total of 43 samples belonging to eight species, covering the two original species of LReR (*L. sinense* and *L. jeholense*) and seven potential substitutes (*L. tenuissimum*, *L. pteridophyllum*, *L. acuminatum*, *S. suave*, *M. yunnanensis*, and *C. vaginatum*), were collected from fields. Voucher specimens were deposited in the Kunming Institute of Botany, Chinese Academy of Sciences (KUN), and Chengdu Institute of Biology, Chinese Academy of Sciences (CDBI) (Supplementary Table S1). To determine possible infraspecific molecular variation, each species was represented by at least two individuals. Furthermore, 25 commercial “Gao-ben” products were purchased from different medicinal markets in China (Supplementary Table S1).

### Laboratory Protocols

Before DNA extraction, the surface of the medicinal materials was wiped with 75% ethanol, then ground into powder with a grinder. Genomic DNA was extracted using the modified CTAB procedure of Doyle and Doyle (1987). The primers BEL-2 (5′-GATGCGGAGATTGGCCCCCCGTGC -3′) and BEL-3 (5′-GACGCTTCTCCAGACTACAAT -3′) were used to amplify the complete ITS2 region (Chiou et al., 2007). The PCR parameters were as follows: Initial denaturation for 3 min at 94°C, followed by 36 cycles of denaturation (94°, 45 s), annealing (55°C, 1 min) and extension (72°C, 2 min), and a final extension for 7 min at 72°C. Purified PCR products were sequenced in both directions with the primers used for PCR amplification on an ABI 3730 automated sequencer (Applied Biosystems, Foster City, CA, United States) in Sangon Biotech Corporation (Shanghai, China).

### Data Analysis

Newly generated sequences were initially edited and assembled using SeqMan of the DNASTAR 5.01 software package (DNASTAR, Inc., Madison, United States). The ITS2 region was annotated using the Hidden Markov Model (HMM) (Keller et al., 2009) to delete the conserved 5.8 and 28S sections (Koetschan et al., 2012). ITS2 secondary structures of all investigated taxa were predicted by homology modeling in the ITS2 Database (identity matrix and 75% threshold for the helix transfer^1^) (Koetschan et al., 2012). This method usually results in several alternative folding patterns for the same ITS2 sequence. The true folding pattern corresponds to the secondary structure model of Mai and Coleman, and was well supported by compensatory base changes (CBCs) and hemi compensatory base changes (hCBCs) revealed by comparisons among related taxa (Mai and Coleman, 1997). Sequences with homologous structure were automatically and synchronously aligned using 4SALE 1.7 (Seibel et al., 2006, 2008). Genetic distances were calculated according to the kimura-2-parameter (K2P) model using MEGA 7.0 software (Kumar et al., 2016). A neighbor-joining (NJ) tree was constructed and bootstrap tests were performed using 1000 replicates to separate the sampled species via MEGA 7.0 (Kumar et al., 2016). CBCs are substitutions in two positions that retain pairing, i.e., G = C ↔ C = G, A = U ↔ U = A. The proposed ITS2 secondary structure was examined for CBCs with the CBCAnalyzer option (Wolf et al., 2005) implemented in 4SALE, whereas hCBCs (pair ↔ non-pair, i.e., G = C ↔ G = U) were observed manually.

## Results

### Amplification, Sequencing, and Sequences Characteristics

The success rate of the ITS2 PCR amplification and sequencing was 100% (Table 1). All high-quality sequences were submitted to GenBank (Table 1). The ITS2 sequence showed minor length variation across all samples, ranging from 220 bp (*M. yunnanensis*) to 226 (*S. suave*). The length of 15 *L. sinense* and *L. jeholense* individuals was 222 bp, and the average GC content was 54.9%. The ITS2 sequence lengths of *L. acuminatum*, *L. delavayi*, *L. pteridophyllum*, *L. tenuissimum*, *C. vaginatum*, *M. yunnanensis*, and *S. suave* were 223, 223, 223, 223, 222, 220, and 226 bp, respectively. The corresponding average GC content of these adulterants varied from 53.2 to 57.9%. The aligned length of 227 bp exhibited 84 variable sites, a rate of 37.0% (Table 1). Therefore, the ITS2 sequences for the sampled species were relatively variable.

**Table 1:** ITS2 sequence characters of samples.

	ITS2
Amplification efficiency (%)	100
Sequencing efficiency (%)	100
Length of all taxa (bp)	220–226
Aligned length (bp)	227
G+C content range in all taxa (%)	53.2–57.9
Number (and %) of variable sites in all taxa	84 (37%)


### Intra/Interspecific Distance, Barcoding Gap, and NJ Tree

**FIGURE 1:**
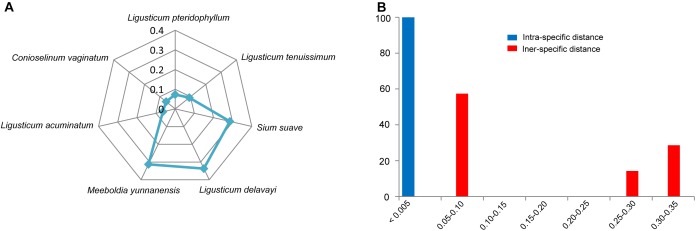
**(A)** Genetic distances from LReR to its adulterants and substitutes. **(B)** Relative distribution of interspecific divergence between LReR and its adulterants and substitutes and intraspecific variation in the ITS2 region using K2P genetic distance.

**FIGURE 2:**
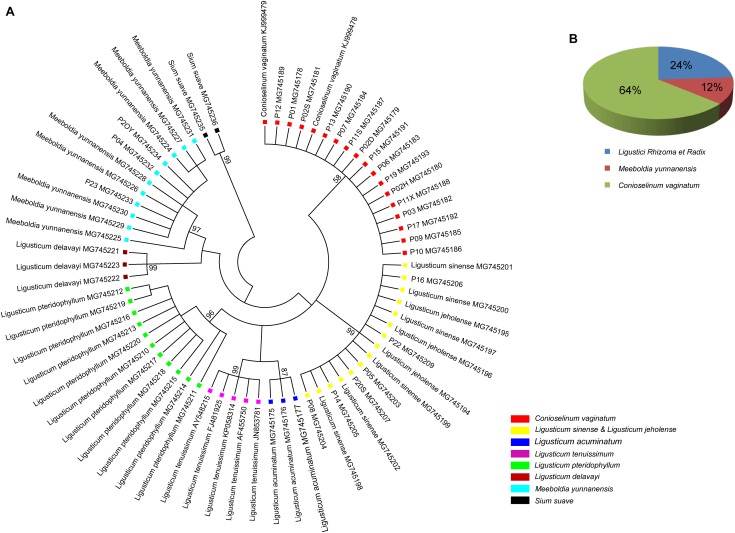
**(A)** NJ tree of LReR and its adulterants and substitutes. Bootstrap scores (1000) are shown (≥50%) for each branch. **(B)** The percentage of the commercial “Gao-ben” products identified by barcode ITS2 in this study.

All individuals of *L. sinense* and *L. jeholense* were identical for ITS2, sharing a single haplotype. According to the K2P model, the average interspecific distance between these and the adulterant species was 0.175, with the maximum interspecific distance being 0.337 from *L. delavayi*, and the minimum being 0.059 from *C. vaginatum* (Table 2 and Figure 1A). The NJ tree showed that 15 individual samples were determined to be *L. jeholense* and *L. sinense* clustered together in a highly supported clade (Bootstrap value = 99) that was separated from the commonly used substitutes and adulterants such as *L. pteridophyllum*, *L. acuminatum*, *L. tenuissimum*, *C. vaginatum*, *S. suave*, *M. yunnanensis*, and *L. delavayi* (Figure 2A). There was therefore a high interspecific variation and an obvious barcoding gap was noted (Figure 1B).

**Table 2:** Analysis of intra-specific variation and inter-specific divergence of the ITS2 sequences.

K2P genetic distances	Range of genetic distances (mean)
Intra-specific distance of LReR	0.000
Inter-specific distance between LReR – *Conioselinum vaginatum*	0.059–0.059 (0.059)
Inter-specific distance between LReR – *L. acuminatum*	0.064–0.064 (0.064)
Inter-specific distance between LReR – *L. pteridophyllum*	0.070–0.076 (0.073)
Inter-specific distance between LReR – *L. tenuissimum*	0.092–0.092 (0.092)
Inter-specific distance between LReR – *Sium suave*	0.282–0.291 (0.286)
Inter-specific distance between LReR – *Meeboldia yunnanensis*	0.309–0.319 (0.313)
Inter-specific distance between LReR – *L. delavayi*	0.337–0.337 (0.337)


Of the 25 commercial “Gao-ben” products, six samples were identified as LReR, accounting for 24%, while the remainder were identified as *M. yunnanensis* (12%), and *C. vaginatum* (64%) (Figure 2B).

### Analysis of the Secondary Structure

**FIGURE 3:**
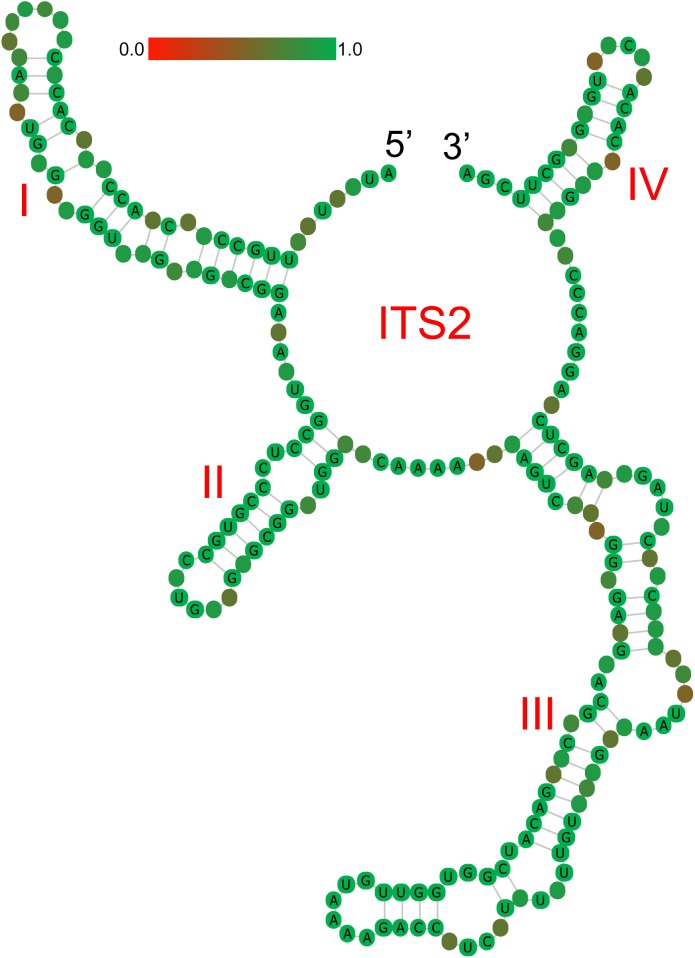
Consensus secondary structure (51%) for all samples without gaps. Helices are numbered I–IV. Sequence conservation is indicated from red/brownish (not conserved) to green (conserved).

ITS2 secondary structures obtained for all species examined fold into the common core structure known for eukaryotes, made up of four helices, the third being the longest (Supplementary Figures S1A–H; Mai and Coleman, 1997; Coleman, 2003, 2007, 2015; Schultz et al., 2005). Figure 3 visualizes a 51% consensus structure. Sequence motifs include a U-U mismatch in helix II, an A-rich conserved spacer between helices II and III, and a UGGU motif 5^′^ side to the apex of helix III (Figure 3). In comparable portions of the secondary structure, most CBCs, and hCBCs observed between LReR and its adulterants are in helices III, I, and II, with a few in helix IV (Table 3). *L. sinense*, *L. jeholense*, *L. tenuissimum*, *L. acuminatum*, *C. vaginatum*, and *L. pteridophyllum* form a group without any CBC in conserved ITS2 regions (i.e., in helices II and III), from which the group of *L. delavayi*, *M. yunnanensis*, and *S. Suave* may be distinguished by the presence of at least one CBC in these regions.

**Table 3:** Occurrence/frequency of CBCs and hCBCs between LReR (*Ligusticum jeholense/L. sinense*) and its adulterants and substitutes.

Taxa	LReR				Summary
	Helix 1	Helix 2	Helix 3	Helix 4	
*L. pteridophyllum*		64/91: G-C → G-U	114/173: U-G → C-G		2 hCBCs
*L. acuminatum*		64/91: G-C → G-U	123/161: U-G → U-A		2 hCBCs
*L. tenuissimum*	20/42: U-G → C-G	64/91: G-C → G-U	123/161: U-G → U-A		3 hCBCs
*L. delavayi*	15/49: G-U → A-U	64/91: G-C → G-U	114/173: U-G → C-G		1 CBCs
		73/82: G-C → G-U	119/164: G-U → G-C		5 hCBCs
			**123/161: C-G → U-A**		
*Conioselinum vaginatum*		64/91: G-C → G-U	123/161: U-G → U-A		2 hCBCs
*Meeboldia yunnanensis*	**15/49: G-C → A-U**	64/91: G-C → G-U	110/177: G-C → G-U	203/220: U-G→ C-G	3 CBCs
			**114/173: U-A → C-G**		3 hCBCs
			**123/161: C-G → U-A**		
*Sium suave*	15/49: G-U → A-U		106/184: U-G → C-G		3 CBCs
	**21/41: U-G → A-U**		110/177: G-C → G-U		5 hCBCs
			115/172: G-U → G-C		
			**122/162: A-U → G-C**		
			**123/161: C-G → U-A**		
			124/160: G-U → G-C		


*Base pairs displaying CBCs are indicated in bolds.*

## Discussion

### Identification Capability of ITS2 for LReR

In Chinese Pharmacopeia, only *L. sinense* and *L. jeholense* are listed as LReR for their very similar chemical compositions and the near identical use and efficacy (Commission, 2015). Morphologically, both species share many similar characters, e.g., ternate-2- or 3-pinnate blade, ultimate segment margins irregularly serrate, and long and reflexed styles. Geographically, *L. jeholense* is in northern China, while *L. sinense* occurs more widely, but does not overlap with *L. jeholense* (Sheh and Watson, 2005). Both our present ITS2 (Table 2) and unpublished data including *psb*A*-trn*H, *mat*K, and *rbc*L show no interspecific variation between these two species. Therefore, we consider that *L. sinense* and *L. jeholense* are close relatives, or the latter represents a vicariant geographical element.

According to the criterion proposed by Coleman (2003) and coworkers, presence/absence of even a single CBC in the conserved areas of helices II and III of ITS2 is associated with incompatibility/inability to hybridize, thus establishing the boundaries between biological species and populations. In contrast, hCBCs in the conserved parts as well as changes in the less conserved regions (e.g., in helices I and IV) do not correlate with interbreeding ability. LReR can be distinguished from *L. delavayi*, *M. yunnanensis*, and *S. suave* by at least one CBC in the conserved ITS2 regions (i.e., in helices II and III) (Table 3 and Supplementary Figures S1F–H). *M. yunnanensis* and *L. delavayi* are widely used as “Huang Gao-ben” in Yunnan Province, whereas *S. suave* has also been sold as “Gao-ben” in medicinal markets. It is difficult to distinguish them from LReR when they are dried, sliced, and shredded, but our ITS2 analysis indicated that they are phylogenetically distant species (Figure 1A, 2A and Table 2). Moreover, these three species can be distinguished from the true LReR by the presence of at least one CBC in the conserved ITS2 regions (i.e., in helices II and III) (Table 3). Results from epidermal analysis (Zhou and Liu, 2018), together with that from cytological evidence (Zhou et al., 2008) and molecular phylogenetics (Zhou et al., 2009), indicated that these are not close relatives of *L. sinense*. We therefore recommend that *L. delavayi*, *M. yunnanensis*, and *S. Suave* should be marketed under their original herbal medicinal name.

Other genetically close relatives of the LReR plant group include *L. pteridophyllum*, L. *tenuissimum*, *L. acuminatum*, and *C. vaginatum* (Figure 1A, 2A and Table 2). *L. pteridophyllum* is another herbal medicine widely used in Yunnan in the name of “Hei Gao-ben” and has been regarded as an adulterant of *Peucedanum praeruptorum* Dunn (Rao et al., 1995). In our analysis, all accessions of *L. pteridophyllum* comprised a strongly supported clade, having a sequence divergence value of 0.073 with LReR (Figure 1A, 2A and Table 2). According to Ye et al. (2004) the chemical composition of *L. pteridophyllum* is similar to LReR, so, considering it as a regional substitute seems to be reasonable. *L. acuminatum* had a sequence divergence value of 0.064 from LReR, for which it is used as a regional substitute in western Sichuan (Sheh and Watson, 2005), so further research is needed to determine whether it can be regarded as an effective “Gao-ben” substitute.

*Conioselinum vaginatum*, known as “Xinjiang Gao-ben,” is found mainly in the Tian and Altai mountains of Xinjiang and western Junggar mountains in central Asia and western Siberia (Sheh and Watson, 2005), and is widely cultivated. Chemical analysis showed that *C. vaginatum* contains 16 compounds, including ligustilide, ferulic acid, and myristic ether, which are the same as in *L. jeholense* (Li, 2013); however, its pharmacological action remains controversial (Dai, 1988; Li et al., 2013). Given that the annual demand for LReR exceeds 3500 tons, which exceeds the natural production capacity (Ding, 2010), research on the pharmacological efficacy of *C. vaginatum* is urgently needed, to determine whether *C. vaginatum* could be an effective substitute.

### Potential Application of ITS2 in the Authentication of Medicinal Materials

The trade in crude drugs has surged globally, generating annual revenues over US $60 billion (Newmaster et al., 2013). There are strong financial incentives for dishonest merchants to use adulterants and substitutes intentionally, leading to treatments not working as advertised, and posing serious risks to the health of consumers (Newmaster et al., 2013). Accurate and rapid species authentication is the best way to combat this. Traditional methods usually require taxonomists, who are few in number, and moreover a fairly complete specimen, meaning they will not work on plant fragments sold as. The ITS2 barcode presented here solves this problem. The ITS2 is region is easy to amplify and sequence, has a short length, and reveals high interspecific variation (Chiou et al., 2007; China Plant Bol Group et al., 2011). While the ITS2 nucleotide sequences evolve quickly, their secondary structures are maintained by certain conserved motifs (Hershkovitz and Zimmer, 1996), which are very useful for sequence alignment (Mai and Coleman, 1997), especially when possible species are spread across many families, as is the case for some Traditional Chinese Medicines (Zhang et al., 2015). Meanwhile, the secondary structures of ITS2 can provide additional molecular morphological characteristics for better species discrimination (Grajales et al., 2007; Gu et al., 2013). Much research has been carried out using ITS2 to regulate the herbal medicine market (Zhang et al., 2015, 2016; Zhao et al., 2015; Zhu et al., 2017), and the current paper shows its effectiveness for LReR or “Gao-ben.” Twenty-five commercial “Gao-ben” samples fell into three clades, corresponding to *L. sinense* + *L. jeholense*, *C. vaginatum*, and *M. yunnanensis* (Figure 2A). Surprisingly, more than half of the samples were derived from *C. vaginatum*. As mentioned above, whether *C. vaginatum* can be substituted for genuine “Gao-ben” is still controversial. Although *M. yunnanensis* is mainly cultivated and used in Yunnan province in the name of “Huang Gao-ben,” its adulteration or substitution can cause confusion in identification and in therapeutic efficacy.

ITS2 has been proved to vary in sequence and secondary structure in a way that highly correlates with species taxonomy. Müller et al. (2007) compared the ITS2 secondary structure of 1373 species to their nearest relatives, and observed that in 93% of cases, if two taxa are different somewhere in their ITS2 by one CBC, they would be classified as different species. This criterion has been less commonly used for herbal medicine authentication. However, in our study, presence of a CBC distinguishes genuine “Gao-ben” from three other species, i.e., *L. delavayi*, *M. yunnanensis*, and *S. suave*, at least the last of which is sometimes sold as “Gao-ben.” So, as a rapid, inexpensive, and informative DNA barcode, ITS2 could be widely used to regulate the herbal medicine market.

## Conclusion

Traditional Chinese medicine is vulnerable to the replacement of the correct and most effective species with others that may be closely or distantly related. ITS2 could be an ideal candidate marker for authentication from both divergences of primary sequences and variations in secondary structures. This method is suitable for the identification of raw medicinal materials, but it is unsuitable for the authentication of heavily processed materials in which DNA degradation frequently occurs. A promising direction suitable for authentication of degraded material would be to combine the next generation sequencing (NGS)-based and species-specific PCR based methods (such as nucleotide signatures). During the process, knowing the secondary structure of ITS2 can help to locate the positions of the short motifs, that is well conserved within the species and develop nucleotide signatures.

## Author Contributions

Z-wL, Y-zG, and JZ collected the samples and carried out the experiments. Z-wL and JZ analyzed the data, conceived and designed the study, and wrote the manuscript. All authors have read and approved the final manuscript.

## Conflict of Interest Statement

The authors declare that the research was conducted in the absence of any commercial or financial relationships that could be construed as a potential conflict of interest.

## Footnotes

1 http://its2.bioapps.biozentrum.uni-wuerzburg.de/

## Supplementary Material

FIGURE S1 Secondary structure of ITS2 in Ligustici Rhizoma et Radix (LReR) and its adulterants and substitutes. **(A)** LReR, **(B)** *L. acuminatum*, **(C)** *L. pteridophyllum*, **(D)** *L. tenuissimum*, **(E)** *Conioselinum vaginatum*, **(F)** *L. delavayi*, **(G)** *Sium suave*, and **(H)** *Meeboldia yunnanensis*. Red and blue arrows show the site of the compensatory base changes (CBCs) and hemi compensatory base changes (hCBCs) between LReR and its adulterants and substitutes, respectively.

TABLE S1 Detailed information of samples used in this study.
