# Supplementary figures and images for: Molecular Authentication of the Medicinal Species of Ligusticum (Ligustici Rhizoma et Radix, “Gao-ben”) by Integrating Non-coding Internal Transcribed Spacer 2 (ITS2) and Its Secondary Structure
Source: Front Plant Sci. 2019 Apr 9;10:429. doi: 10.3389/fpls.2019.00429 (PMC6465525; doi:10.3389/fpls.2019.00429)

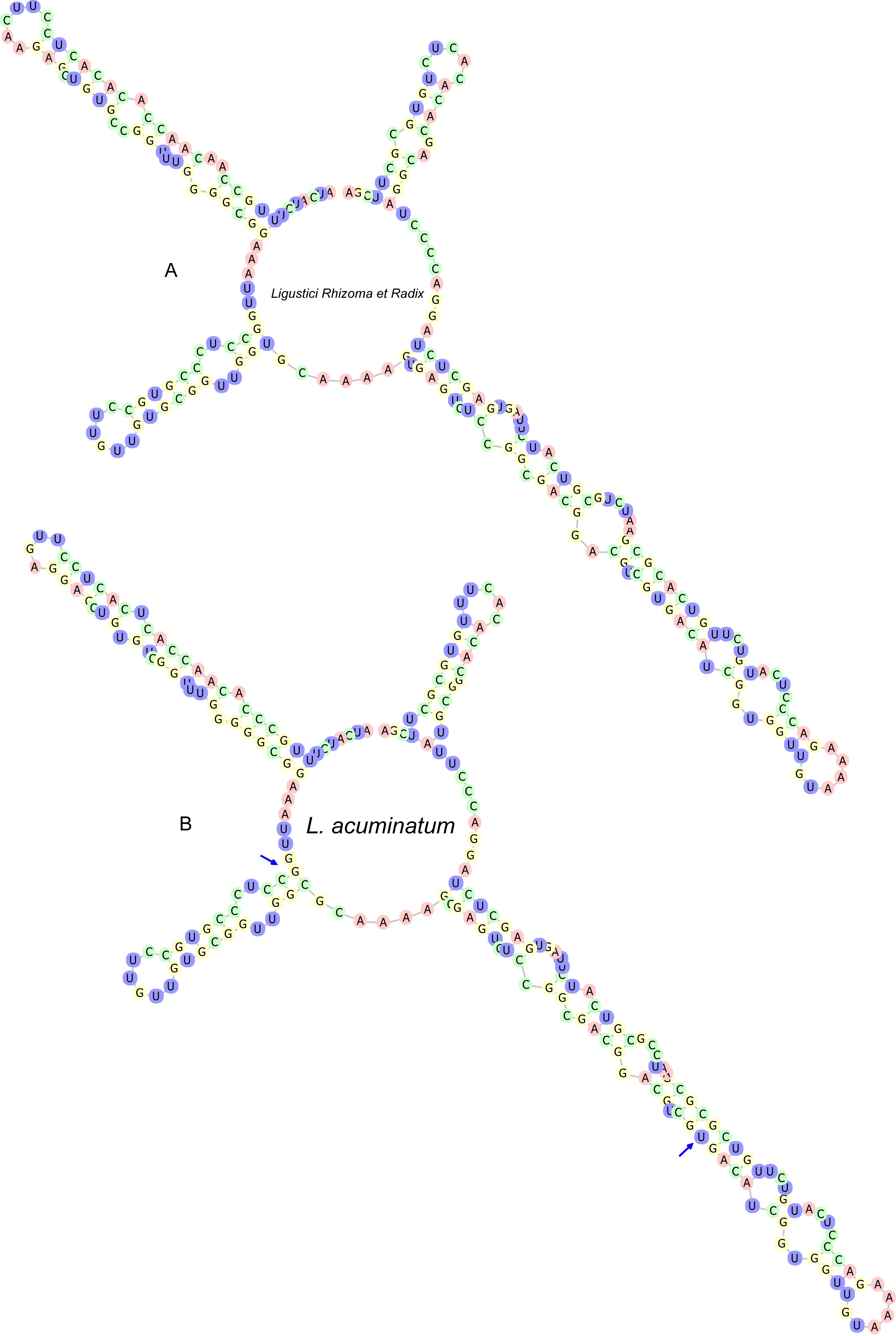

Supplement: FIGURE S1 — Secondary structure of ITS2 in Ligustici Rhizoma et Radix (LReR) and its adulterants and substitutes. (A) LReR, (B) L. acuminatum, (C) L. pteridophyllum, (D) L. tenuissimum, (E) Conioselinum vaginatum, (F) L. delavayi, (G) Sium suave, and (H) Meeboldia yunnanensis. Red and blue arrows show the site of the compensatory base changes (CBCs) and hemi compensatory base changes (hCBCs) between LReR and its adulterants and substitutes, respectively. [file Image_1.TIF]

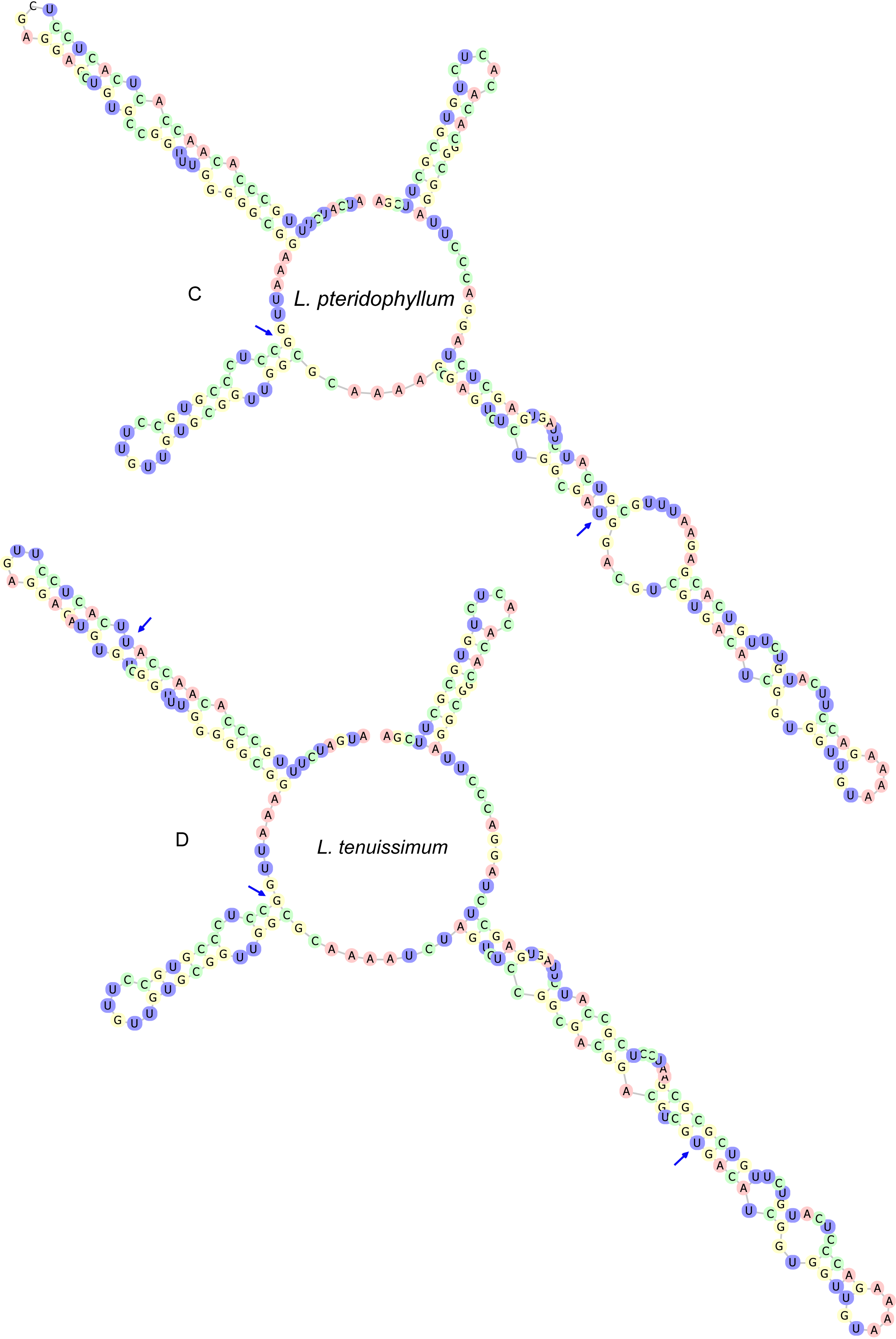

Supplement: Supplementary file 3 [file Image_2.TIF]

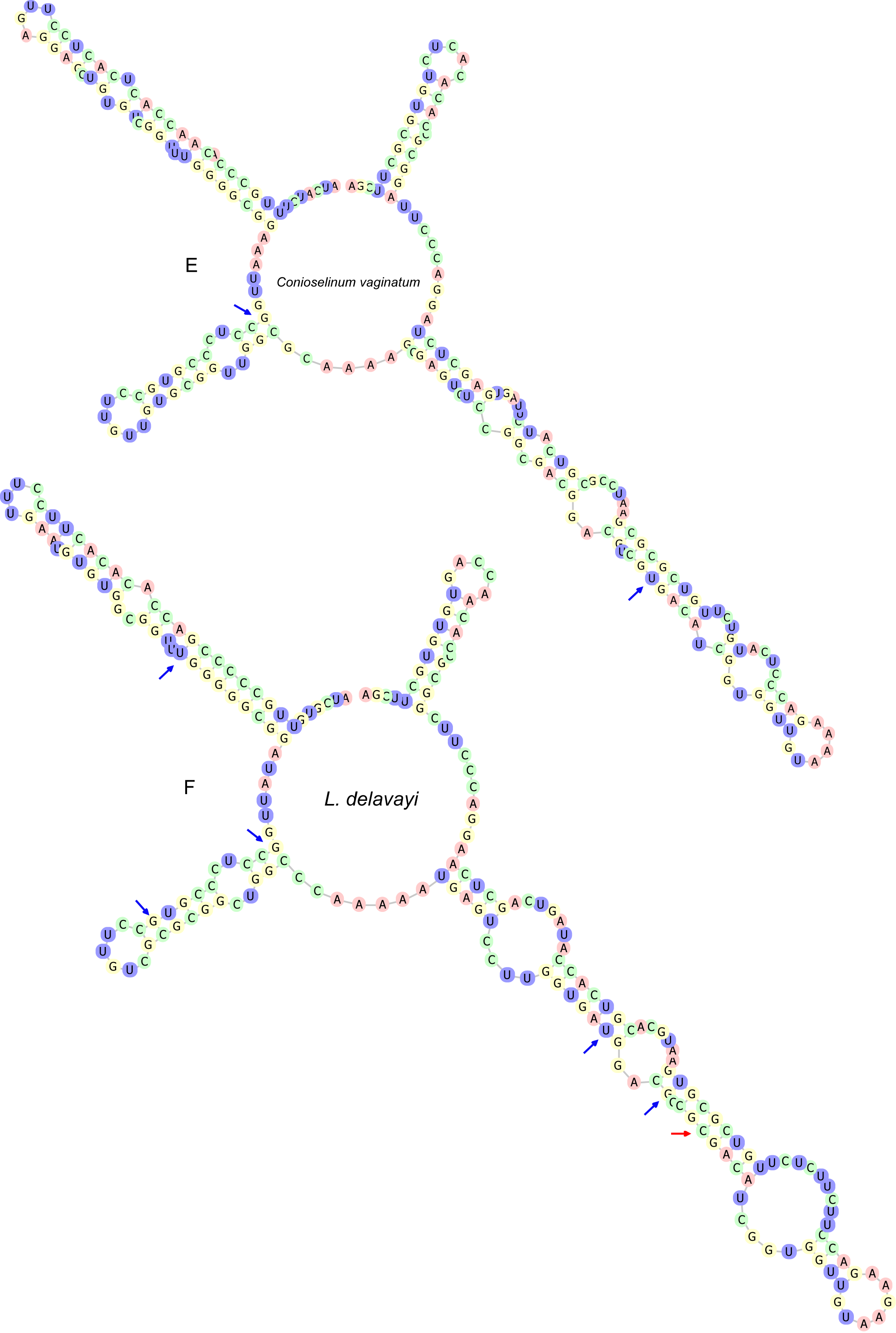

Supplement: Supplementary file 4 [file Image_3.TIF]

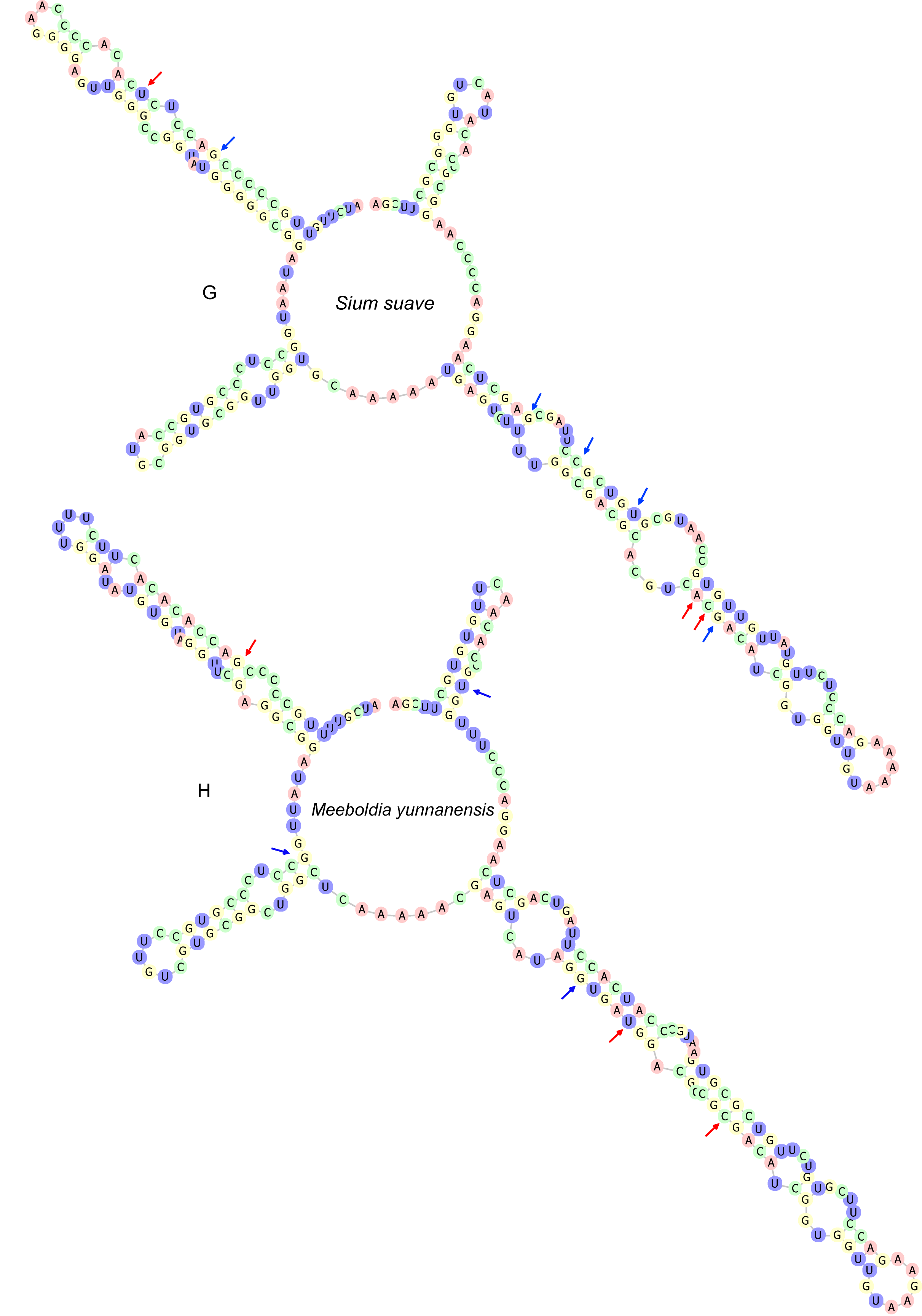

Supplement: Supplementary file 5 [file Image_4.TIF]
