# Supplementary material for: Molecular Authentication of the Medicinal Species of Ligusticum (Ligustici Rhizoma et Radix, “Gao-ben”) by Integrating Non-coding Internal Transcribed Spacer 2 (ITS2) and Its Secondary Structure
Source: Front Plant Sci. 2019 Apr 9;10:429. doi: 10.3389/fpls.2019.00429 (PMC6465525; doi:10.3389/fpls.2019.00429)
Supplement: TABLE S1 — Detailed information of samples used in this study. [file Table_1.DOCX]

**TABLE S1.** Detailed information of samples used in this study.

| **Taxa** | **Voucher No.** | **Locality information** | **Genbank no.** |
| --- | --- | --- | --- |
| S1^#^ | P01 | Haozhou, Anhui | MG745178* |
| S2^#^ | P02D | Huanggang, Hubei | MG745179* |
| S3^#^ | P02H | Huanggang, Hubei | MG745180* |
| S4^#^ | P02S | Huanggang, Hubei | MG745181* |
| S5^#^ | P03 | Chengdu, Sichuan | MG745182* |
| S6^#^ | P04 | Kunming, Yunnan | MG745232* |
| S7^#^ | P05 | Yuncheng, Shanxi | MG745203* |
| S8^#^ | P06 | Hanzhong, Shaanxi | MG745183* |
| S9^#^ | P07 | Jining, Shandong | MG745184* |
| S10^#^ | P08 | Shantou, Guangdong | MG745204* |
| S11^#^ | P09 | Haozhou, Anhui | MG745185* |
| S12^#^ | P10 | Jieyang, Guangdong | MG745186* |
| S13^#^ | P11S | Yulin, Guangxi | MG745187* |
| S14^#^ | P11X | Yulin, Guangxi | MG745188* |
| S15^#^ | P12 | Baoding, Hebei | MG745189* |
| S16^#^ | P13 | Baoding, Hebei | MG745190* |
| S17^#^ | P14 | Nanyang, Henan | MG745205* |
| S18^#^ | P15 | Yancheng, Jiangsu | MG745191* |
| S19^#^ | P16 | Chengdu, Sichuan | MG745206* |
| S20^#^ | P17 | Jinhua, Zhejiang | MG745192* |
| S21^#^ | P19 | Laibin, Guangxi | MG745193* |
| S22^#^ | P20Y | Kunming, Yunnan | MG745234* |
| S23^#^ | P20S | Kunming, Yunnan | MG745207* |
| S24^#^ | P22 | Chengdu, Sichuan | MG745209* |
| S25^#^ | P23 | Lufeng, Yunnan | MG745233* |
| *Conioselinum vaginatum* | - | GenBank | KJ999479 |
|  | - | GenBank | KJ999478 |
| *Ligusticum acuminatum* |  | Hongyuan, Sichuan | MG745175* |
|  | CDBI 0093687 | Maerkang, Sichuan LZ1141 | MG745176* |
|  | CDBI 0102313 | Sichuan precise collection site unknown | MG745177* |
| *L. delavayi* | KUN 0462952 | Lijiang, Yunnan | MG745221* |
|  | LZ151006 | Heqing, Yunnan | MG745222* |
|  | KUN 0462949 | Zhongdian, Yunnan | MG745223* |
| *L. jeholense* | [KUN 0463049](http://www.cvh.ac.cn/spm/KUN/0463049) | Neiqiu, Hebei | MG745194* |
|  | [KUN 0463051](http://www.cvh.ac.cn/spm/KUN/0463051) | Xinglong, Hebei | MG745195* |
|  | LJ5 | Institute of Botany, Beijing | MG745196* |
| *L. pteridophyllum* | Z1510 | Lijiang, Yunnan | MG745210* |
|  | KUN 0463100 | Huidong, Sichuan | MG745211* |
|  | [KUN 0463129](http://www.cvh.ac.cn/spm/KUN/0463129) | Lushui, Yunnan | MG745212* |
|  | KUN 0463103 | Daocheng, Sichuan | MG745213* |
|  | KUN 0463131 | Xuanwei, Yunnan | MG745214* |
|  | LZ1105 | Kangding, Sichuan | MG745215* |
|  | LZ0951 | Eryuan, Yunnan | MG745216* |
|  | W20100925 | Dali, Yunnan | MG745217* |
|  | 20160655 | Muli, Sichuan | MG745218* |
|  | 20160633 | Yanyuan, Sichuan | MG745219* |
|  | 20160649 | Yanyuan, Sichuan | MG745220* |
| *L. sinense* | LZ151002 | Heqing, Yunnan | MG745197* |
|  | [KUN 0463210](http://www.cvh.ac.cn/spm/KUN/0463210) | Suichuan, Jiangxi | MG745198* |
|  | [KUN 0463207](http://www.cvh.ac.cn/spm/KUN/0463207) | Xingshan, Hubei | MG745199* |
|  | [KUN 0463214](http://www.cvh.ac.cn/spm/KUN/0463214) | Taibai, Shanxi | MG745200* |
|  | 20160607 | Yanshan, Yunnan | MG745201* |
|  | LZ1169 | Maerkang, Sichuan | MG745202* |
| *L. tenuissimum* | - | GenBank | FJ481925 |
|  | - | GenBank | JN853781 |
|  | - | GenBank | KP058314 |
|  | - | GenBank | AY548215 |
|  | - | GenBank | AF455750 |
| *Meeboldia yunnanensis* | LZ1518 | Luquan, Yunnan | MG745224* |
|  | [KUN 0463250](http://www.cvh.ac.cn/spm/KUN/0463250) | Daocheng, Sichuan | MG745225* |
|  | KUN 0463261 | Heqing, Yunnan | MG745226* |
|  | KUN 0463257 | Songming, Yunnan | MG745227* |
|  | KUN 0463259 | Kunming, Yunnan | MG745228* |
|  | KUN 0463277 | Dali, Yunnan | MG745229* |
|  | C152 | Dayao, Yunnan | MG745230* |
|  | LZ0942 | Yuanmou, Yunnan | MG745231* |
| *Sium suave* | KUN 1248964 | Zhanghua, Taiwan | MG745235* |
|  | S6 | Institute of Botany, Beijing | MG745236* |

^#^ Commercial “Gao-ben” products purchased from different medicinal markets in China

*newly acquired sequences in this study
